# Medication adherence, biological and lifestyle risk factors in patients with myocardial infarction: a ten-year follow-up on socially differentiated cardiac rehabilitation

**DOI:** 10.1080/02813432.2019.1608046

**Published:** 2019-05-23

**Authors:** Kathrine Hald, Finn Breinholt Larsen, Kirsten Melgaard Nielsen, Lucette Kirsten Meillier, Martin Berg Johansen, Mogens Lytken Larsen, Bo Christensen, Claus Vinther Nielsen

**Affiliations:** a Section for Clinical Social Medicine and Rehabilitation Department of Public Health, Aarhus University, Aarhus, Denmark;; b DEFACTUM, Social and Health Services and Labour Market, Central Denmark Region, Aarhus, Denmark;; c Department of Cardiology, Aarhus University Hospital, Aarhus, Denmark;; d Unit of Clinical Biostatistics, Aalborg University Hospital, Aalborg, Denmark;; e Danish Centre for Inequality in Health, Department of Cardiology, Aalborg University Hospital, Aalborg, Denmark;; f Section for General Medical Practice, Department of Public Health, Aarhus University, Aarhus, Denmark

**Keywords:** Cardiovascular diseases, rehabilitation, socioeconomic factors, secondary prevention, medication adherence, risk factor management

## Abstract

**Objective:** There is strong evidence that medication adherence and lifestyle changes are essential in patients undergoing secondary cardiovascular disease prevention. Cardiac rehabilitation (CR) increases medication adherence and improves lifestyle changes. Patients with cardiac diseases and a low educational level and patients with little social support are less responsive to improve medication adherence and to adapt lifestyle changes. The aim of the present study was to investigate the long-term effects of a socially differentiated CR intervention on medication adherence as well as changes in biological and lifestyle risk factors at two- five- and ten-year follow-up.

**Design:** A prospective cohort study.

**Setting:** The cardiac ward at Aarhus University Hospital, Denmark.

**Intervention:** A socially differentiated CR intervention in addition to the standard CR program.

**Subjects:** Patients admitted with first-episode myocardial infarction between 2000 and 2004, *N =* 379. Patients were defined as socially vulnerable or non-socially vulnerable according to their educational level and extent of social network.

**Main outcome measures:** Primary outcome was medication adherence to antithrombotics, beta-blockers, statins and angiotensin-converting enzyme inhibitors. Secondary outcomes were biological and lifestyle risk factors defined as; total cholesterol, low-density lipoprotein cholesterol, high-density lipoprotein cholesterol, glycated hemoglobin, blood pressure and smoking status.

**Results:** No significant long-term effect of the intervention was found.

**Conclusions:** The results indicate a non-significant effect of the intervention. However, it was found that equality in health was improved in the study population except concerning smoking. General practitioners manage to support the long-term secondary cardiovascular disease prevention in all patients regardless of social status.

**Key points**
- The socially differentiated intervention did not significantly improve medication adherence or biological and lifestyle risk factors.
- Despite the non-significant effect of the intervention, equality in health was improved except concerning smoking.
- General practitioners managed to support the long-term secondary cardiovascular disease prevention in all patients regardless of social status.

## Introduction

The '2016 European Guidelines on cardiovascular disease prevention in clinical practice' emphasizes that medication adherence and lifestyle changes are essential in secondary cardiovascular disease prevention [1]. A core component of this is cardiac rehabilitation (CR), which is a structured and multidisciplinary intervention [2]. Several studies have shown that CR increases medication adherence and adherence to lifestyle changes [3–8]. Long-term adherence to medication and lifestyle is crucial and associated with a lower risk of mortality and recurrent events [1,2,9,10]. Key strategies to improve adherence include a strong relationship between clinician and patient, a safe transition from hospital to primary care and communication skills to ensure the patient’s understanding of the risks of non-adherence [11].

Patients with a low educational level and little social support are less responsive to achieving medication and lifestyle changes when diagnosed with cardiovascular disease (CVD) [12–16]. Even in countries with equal and free access to health care, social inequalities are observed, implying that new initiatives are needed in secondary cardiovascular disease prevention [17].

In a Danish socially differentiated CR intervention performed from 2000 to 2004, the focus was on minimizing social inequality in patients diagnosed with myocardial infarction (MI). At one-year follow-up, socially vulnerable patients receiving the intervention had a significantly better medication adherence, a significantly better lipid profile and a significantly lower systolic blood pressure (BP) when compared to socially vulnerable patients receiving standard CR [18].

The aim of the present study was to investigate the long-term effects of the above mentioned socially differentiated CR intervention on medication adherence as well as changes in biological and lifestyle risk factors at two- five- and ten-year follow-up.

## Material and methods

### Design and study participants

The study was designed as a prospective cohort study and conducted from 1 April 2000 to 31 December 2004. The study population was enrolled at admission to hospital and follow-up was conducted at two, five and ten years. The patients entering the study were all <70 years, admitted at Aarhus University Hospital, Denmark and diagnosed with first episode of MI. Patients were excluded if they suffered from severe comorbidities such as stroke, dementia, mental disorders (not depression or anxiety), retardation or severe alcohol abuse [18,19].

The study was divided into two phases which consisted of two years observation of clinical practice regarding standard CR from 2000 to 2002 and followed by a two-year intervention from 2002 to 2004. A total of 508 patients were admitted with first episode MI and of these 379 patients (75%) who were offered and attended CR were included in the study and provided written informed consent [18,19].

When entering the study, patients were defined as socially vulnerable or non-socially vulnerable. If patients had a low educational level or lived alone they were defined as socially vulnerable. 78 patients admitted between 2000 and 2002 and 130 patients admitted between 2002 and 2004 met the above criteria and were categorized as socially vulnerable. The remaining 171 patients, 55 patients admitted between 2000 and 2002 and 116 patients admitted between 2002 and 2004, were categorized as non-socially vulnerable. Further details of the definition have been described previously [18,19].

### Intervention

All 379 patients received standard CR in accordance with international guidelines [1]. In CR phase I from hospital admission to discharge, patients received medical and acute surgical treatment. In CR phase II from discharge and the next 12 weeks, patients had three consultations with a doctor, four consultations with a nurse, two consultations with a dietician and participated in 12-week exercise program. In CR phase III, patients were referred to general practice and informed about relevant activities in The Danish Heart Association and in the municipal sector [18,19].

The 130 patients categorized as socially vulnerable and admitted between 2002 and 2004 received an expanded CR intervention in addition to the standard program. The expanded CR intervention was two week longer in phase II and contained an extra consultation with a nurse. The patients played an active part in designing their own rehabilitation plan which was sent to their general practitioner. The patients were referred to a phase III start-up consultation in general practice which was based on their individual rehabilitation plan. The patients were referred to activities in The Danish Heart Association and the municipal sector. Moreover, patients received a follow-up telephone call from a nurse three months after completing phase II CR [18,19].

### Outcomes

Primary outcome was medication adherence to antithrombotics, beta-blockers, statins and angiotensin-converting enzyme inhibitors (ACE inhibitors). Secondary outcomes were biological and lifestyle risk factors defined as; total cholesterol, low-density lipoprotein (LDL) cholesterol, high-density lipoprotein (HDL) cholesterol, glycated hemoglobin (HbA1c), blood pressure and smoking status.

### Data collection and measures

In Denmark, citizens are assigned a unique personal 10-digit number, which was used in the data collection. Information on medication adherence was obtained from The Danish National Prescription Register [20]. The database contains information on all prescription drugs sold in Denmark. Medication adherence was defined as the purchase of at least one prescription every year of the follow-up period with the specific ATC-codes B01AC04, B01AC06, B01AC56 for anti-thrombotics, C07AA, C07AB for beta-blockers, C10AA, C10AB, C10AC, C10AD, C10AX09, C10BA for statins and C09AA, C09BB, C09CA, C09D for ACE inhibitors.

Information on total cholesterol, LDL-cholesterol, HDL-cholesterol, triglyceride and HbA1c was obtained from a local laboratory database 'LABKA' containing information on results of all blood tests performed in both the primary and secondary sector in the region where Aarhus University Hospital is located [21]. Cholesterol and triglyceride were measured as mmol/L and HbA1c was measured as mmol/mol. The value for each of the laboratory outcomes at the time of follow-up was an average value computed from all the values obtained in the follow-up period.

Information on blood pressure and smoking status was collected through a questionnaire sent to the patient's general practitioner. In Denmark, patients diagnosed with MI are entitled to an annual chronic care consultation in general practice [22]. The general practitioner was informed about the patient's admission date and provided information on blood pressure and smoking status at the annual consultation. Blood pressure was measured as mm/Hg and smoking status was reported as smoker/non-smoker. The questionnaire data were typed into a data documentation program by two different evaluators. All answers were assessed and if any dissimilarities occurred, the questionnaires were reevaluated by both evaluators.

### Statistical analysis

The baseline characteristics of patients in each group are described using frequencies and percentages or means and standard deviations as appropriate. Evaluations of the primary and secondary outcomes were performed as visualizations of the outcome measures during follow-up, and as statistical tests at follow-up between socially vulnerable patients receiving the standard CR versus the expanded CR. To evaluate the potential differences between the two calendar periods, a supplemental analysis of the non-socially vulnerable patients in the two corresponding time periods was also conducted. All data were based on yearly survivors in the study population. Data on medication adherence were shown as yearly proportions of patients who had redeemed at least one prescription for each drug. The 95% confidence intervals (CIs) of the proportions at follow-up were also calculated. Comparisons were evaluated using a chi-square test of independence.

The biomarker data were based on all blood samples collected through LABKA and shown as medians in visualizations and compared by calculating differences in means with 95% CIs and using a *t* test to test for equality of means. The questionnaire data were summarized and evaluated by calculating means and proportions with the corresponding tests as described for the medication and biomarker data. All data management and analyses were performed using Stata/MP 14.2, and *p*-values less than .05 were considered statistically significant.

## Results

Baseline characteristics of the study population can be seen in Table 1. The mean age was 57 years and approximately three out of four were male. As a result of the definition of socially vulnerable patients had a lower educational level and a higher fraction was living alone. Total cholesterol, fasting blood glucose, body mass index and smoking status were quite similar in all groups, except in the group of socially vulnerable patients admitted between 2000 and 2002 who had higher values and smoked more. Patients admitted between 2000 and 2002 were prescribed ACE inhibitors and statins less often than patients admitted between 2002 and 2004 regardless of social status.

**Table 1.** Baseline characteristics of 379 patients admitted with first-episode myocardial infarction receiving socially differentiated cardiac rehabilitation.

	Socially vulnerable participants			Non-socially vulnerable participants		
	Rehabilitation type *N* Time period			Rehabilitation type *N* Time period		
	Standard Rehabilitation	Expanded Rehabilitation		Standard Rehabilitation	Standard Rehabilitation	
	*N* = 78	*N* = 130		*N* = 55	*N* = 116	
	2000–2002	2002–2004	*p* Value	2000–2002	2002–2004	*p* Value
Age at admission, years	56 (8.2)	55 (8.5)	.65	60 (7.6)	57 (73)	.02
Gender, male	57 (73)	93 (71)	.81	42 (76)	94 (81)	.48
Education level (DUN)	3.2 (1.2)	3.3 (1.4)	.66	4.8 (1.1)	4.8 (1.2)	.79
Living alone	27 (35)	51 (39)	.51	0	0	–
Total cholesterol, mmol/L	5.7 (1.5)	5.2 (1.0)	.00	5.2 (0.9)	5.2 (0.9)	.88
LDL cholesterol, mmol/L	3.5 (1.0)	3.2 (0.9)	.02	3.3 (0.9)	3.2 (0.8)	.58
HDL cholesterol, mmol/L	1.2 (0.3)	1.2 (0.3)	.13	1.3 (0.3)	1.3 (0.4)	.89
Triglyceride, mmol/L	2.0 (1.1)	1.7 (1.0)	.02	1.5 (1.0)	1.7 (0.9)	.25
Fasting blood glucose, mmol/L	7.5 (4.6)	6.9 (2.8)	.25	6.8 (3.3)	6.7 (2.0)	.69
Body Mass Index	27.3 (4.4)	26.3 (4.1)	.10	26.4 (4.0)	26.5 (3.1)	.77
Prescribed beta-blocker	71 (91)	116 (89)	.67	49 (89)	107 (92)	.50
Prescribed ACE-inhibitor	24 (31)	55 (42)	.09	20 (36)	49 (42)	.46
Prescribed statin	20 (26)	99 (76)	.00	10 (18)	104 (90)	.00
Prescribed anti-thrombotics	72 (92)	126 (97)	.13	47 (86)	112 (97)	.01
Current smoker	59 (76)	83 (64)	.28	34 (62)	60 (52)	.29

Patients are divided into groups based on social vulnerability and time period of admission.

### Medication adherence

**Figure 1.**
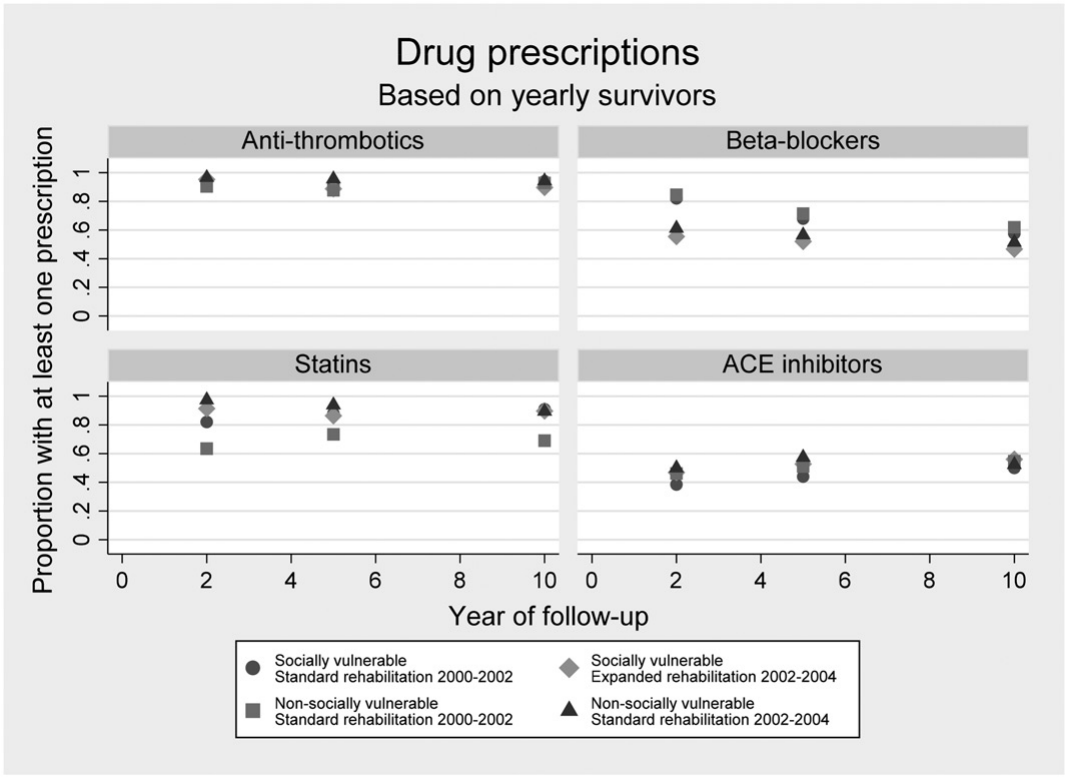
Proportions of patients redeeming at least one prescription for anti-thrombotics, beta-blockers, statins and ACE inhibitors each year after first-episode myocardial infarction admission by groups of social vulnerability and calendar period of admission. Proportions are based on all patients with a first admission at Aarhus University Hospital, Denmark between 2000 and 2004 (*N* = 379) who survived each year of follow-up.

As indicated in Figure 1, adherence to anti-thrombotics during the ten-year follow-up was higher than 80%. This was also the case in relation to statins with one exception, as the non-socially vulnerable patients admitted between 2000 and 2002 showed a steady above 60% adherence during the ten-year follow-up. Adherence to beta-blockers was higher in the groups admitted between 2000 and 2002 than in the groups admitted between 2002 and 2004 irrespective of whether the patients were categorized as socially vulnerable or not. Adherence to ACE inhibitors was around 40–60% in all groups throughout the 10-year follow-up.

In relation to anti-thrombotics and ACE-inhibitors, no significant differences in adherence were seen between the socially vulnerable patients at two-, five- and ten-year follow-up. In relation to beta-blockers, a significant difference was seen at two- and five-year follow-up (*p*-values .00 and .02), showing that significantly more socially vulnerable patients receiving standard CR redeemed at least one prescription on beta-blockers in each of the follow-up years. No significant difference was seen at ten-year follow-up. In relation to statins, a significant difference was seen at two-year follow-up (*p*-value .04). Significantly more socially vulnerable patients receiving the expanded CR purchased at least one prescription on statins in the follow-up year. No significant differences were seen at five- or ten-year follow-up (Table 2). As visualized in Figure 1, some of the same tendencies were observed in the group of non-socially vulnerable patients.

**Table 2.** Assessment of medication adherence and biological and lifestyle risk factors among socially vulnerable patients admitted between 2000 and 2002 (*N* = 78) and between 2002 and 2004 (*N* = 130) at Aarhus University Hospital, Denmark with first-episode myocardial infarction who participated in socially differentiated cardiac rehabilitation intervention and who were followed-up at two, five and ten years.

	Socially vulnerable participants						
		2000–2002		2002–2004			
	Year of follow-up	Proportion* Mean**	*N*	Proportion* Mean**	*N*	Ratio* Difference**	*p* value
Anti-thrombotic	2	0.90*	78	0.95*	128	1.1* (0.9–1.1)	.37
	5	0.92*	75	0.89*	125	1.0* (0.9–1.1)	.46
	10	0.94*	66	0.90*	107	1.0* (0.9–1.0)	.33
Beta-blockers	2	0.82*	78	0.55*	128	0.7* (0.6–0.8)	.00
	5	0.68*	75	0.52*	125	0.8 *(0.6–1.0)	.02
	10	0.58*	66	0.47*	107	0.8* (0.6–1.1)	.16
Statins	2	0.82*	78	0.91*	128	1.1* (1.0–1.3)	.04
	5	0.88*	75	0.86*	125	1.0* (0.9–1.1)	.74
	10	0.91*	66	0.90*	107	1.0* (0.9–1.1)	.79
ACE inhibitors	2	0.38*	78	0.47*	128	1.2* (0.9–1.7)	.23
	5	0.44*	75	0.53*	125	1.2* (0.9–1.6)	.22
	10	0.50*	66	0.56*	107	1.1* (0.8–1.5)	.43
Total Cholesterol	2	5.1**	78	4.4**	130	−0.6 **(–0.8 to −0.4)	.00
	5	4.7**	74	4.3**	116	−0.4 **(–0.6 to −0.2)	.00
	10	4.3**	68	4.2**	105	−0.1** (–0.3–0.2)	.51
HDL Cholesterol	2	1.2**	78	1.3**	130	0.1** (0.1− 0.2)	.01
	5	1.3**	74	1.3**	114	0.0 **(−0.1–0.1)	.95
	10	1.2**	68	1.3**	103	0.1 **(−0.0–0.2)	.11
LDL Cholesterol	2	3.0**	77	2.5**	130	−0.5 **(−0.7 to −0.4)	.00
	5	2.6**	74	2.3**	111	−0.3 **(−0.4 to −0.1)	.00
	10	2.4**	68	2.3**	102	−0.1** (−0.3–0.1)	.37
Triglyceride	2	2.0**	78	1.5**	130	−0.4 **(−0.7 to −0.2)	.00
	5	1.8**	74	1.6**	112	−0.2 **(−0.4 to −0.0)	.04
	10	1.7**	68	1.6**	103	−0.03** (−0.3–0.2)	.80
HbA1c	2	50.7**	30	42.3**	117	−8.4 **(−12.6 to −4.2)	.00
	5	52.1**	40	45.7**	71	−6.4 **(−11.3 to −1.4)	.01
	10	48.9**	43	44.9**	93	−4.0** (−8.1–0.1)	.06
Systolic blood pressure	2	131.8**	53	131.4**	93	−0.4** (−6.0–5.1)	.89
	5	132.7**	54	132.6**	96	−0.1** (−6.8–6.5)	.97
	10	134.1**	55	132.4**	92	−1.7** (−7.5–4.2)	.57
Diastolic blood pressure	2	79.5**	53	79.0**	93	−0.5** (−3.9–2.9)	.77
	5	78.3**	54	79.0**	96	0.7** (−2.7–4.1)	.68
	10	80.0**	55	79.4**	92	−0.5** (−4.2–3.2)	.78
Smoking status	2	0.52*	33	0.46*	70	0.9* (0.6–1.4)	.58
	5	0.59*	39	0.47*	68	0.8* (0.6–1.2)	.23
	10	0.37*	43	0.37*	75	1.0* (0.6–1.6)	.98

Values are based on yearly survivors and on available data from registers and questionnaires.

### Biological and lifestyle risk factors

**Figure 2.**
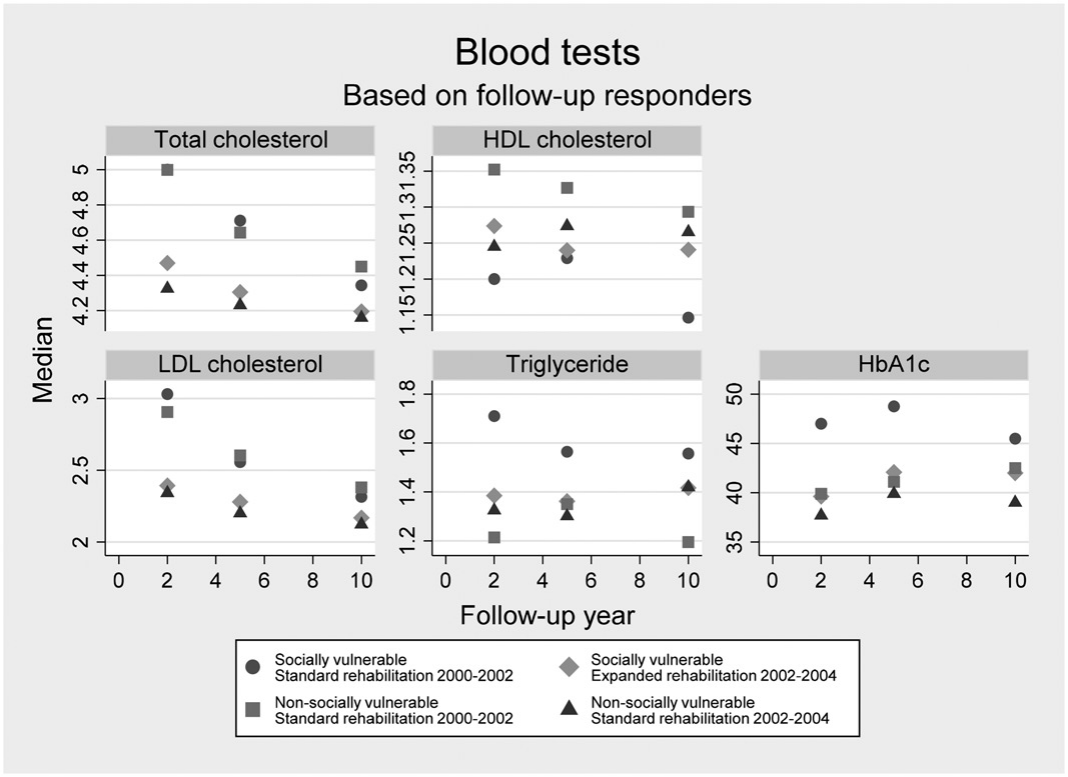
Median values of blood test results among patients each year after first-episode myocardial infarction by groups of social vulnerability and calendar period of admission. Medians are based on data from all patients with a first admission at Aarhus University Hospital, Denmark between 2000 and 2004 (*N* = 379) who have a valid register-based value of each type of blood test within each year of follow-up.

As seen in Figure 2, the levels of the blood tests were acceptable. In general, the patients admitted between 2000 and 2002 had less acceptable blood test levels in the first years after baseline regardless of being categorized as socially vulnerable or not. This effect was less clear during the last part of the follow-up.

Socially vulnerable patients receiving the expanded CR intervention had significantly lower total cholesterol (*p*-values .00, .00), LDL cholesterol (*p*-values .00, .00), triglyceride (*p*-values .00, .04) and HbA1c levels (*p*-values .00, .01) at two- and five- year follow-up. No significant differences were seen at ten-year follow-up. Socially vulnerable patients receiving the expanded CR intervention showed significantly higher HDL cholesterol at two-year follow-up (*p*-value .01). No significant differences were seen at five- and ten-year follow-up (Table 2). As visualized in Figure 2, some of the same tendencies were present in the group of non-socially vulnerable patients. However, no significant differences were seen in relation to triglyceride.

Of the 379 forwarded questionnaires, 301 were returned by the general practitioners (response rate 79%). Not all returned questionnaires were filled out completely. The response rate in the group of socially vulnerable patients admitted between 2000 and 2002 was 77% and it was 81% in the group of socially vulnerable patients admitted between 2002 and 2004 (*p*-value .50). The response rate in the group of non-socially vulnerable patients admitted between 2000 and 2002 was 69% and it was 84% in the group of non-socially vulnerable patients admitted between 2002 and 2004 (*p*-value .02).

**Figure 3.**
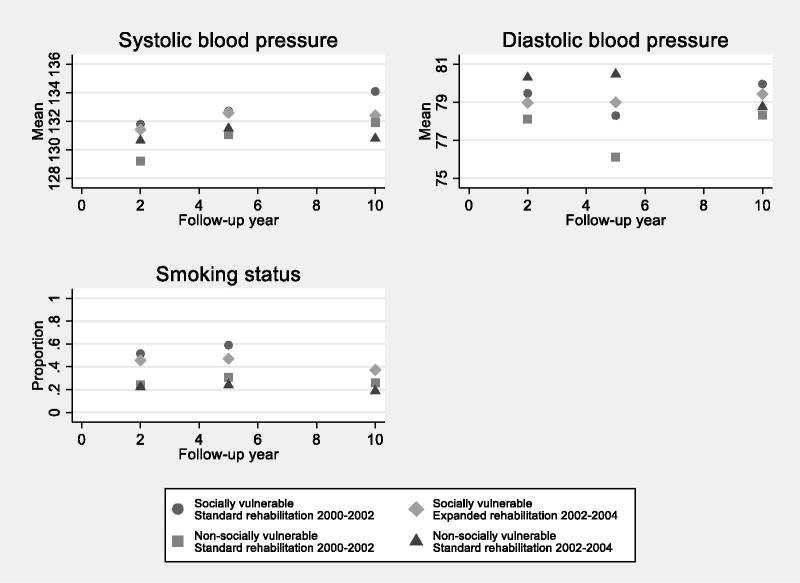
Mean values and proportions of blood pressure and smoking status among patients with first-episode myocardial infarction admission by groups of social vulnerability and calendar period of admission. Values are based on questionnaire data from general practitioners of all patients with a first admission at Aarhus University Hospital, Denmark between 2000 and 2004 (*N* = 379) with a valid questionnaire response at each time of follow-up.

As seen in Figure 3, patients had acceptable mean blood pressure levels regardless of being categorized as socially vulnerable or not. No mean blood pressure levels of more than 135/85 mm/Hg were seen. No significant differences in systolic or diastolic blood pressure were seen between socially vulnerable patients receiving the standard CR and socially vulnerable patients receiving the expanded CR during the ten-year follow-up (Table 2). As visualized in Figure 3, some of the same tendencies were seen in the group of non-socially vulnerable patients.

Figure 3 shows that a higher proportion of socially vulnerable patients than non-vulnerable patients smoked . The proportion of smokersdecreased during the first two years after admission in all groups when compared to baseline data (Table 1). The proportion of smokers increased from two to five years after admission and declined again in each of the groups during the remaining part of the ten-year follow-up. No significant differences in smoking status were seen between socially vulnerable patients receiving the standard CR and socially vulnerable patients receiving the expanded CR during the ten-year follow-up (Table 2). As visualized in Figure 3, the same tendency was present in the group of non-socially vulnerable patients.

## Discussion

The present study investigated the long-term effects of a socially differentiated CR intervention on medication adherence, biological and lifestyle risk factors at two- five- and ten-year follow-up. Overall, no significant long-term effects were found. However, significantly more patients categorized as socially vulnerable receiving the expanded CR redeemed at least one prescription on statins at the two-year follow-up. Also, socially vulnerable patients receiving the expanded CR had significantly lower levels of total cholesterol, LDL cholesterol, triglyceride and HbA1c at two- and five-year follow-up and a significantly higher level of HDL cholesterol at two-year follow-up.

### Strengths and weaknesses

A strength of the study is that the majority of the data are retrieved from Danish registers, which can be assumed to provide an almost complete follow-up. The risk of selection bias in data collected through questionnaires should, however, always be considered. As the questionnaires were filled out by the patients' general practitioners we have no reasons to believe that selection bias was present. Also, no significant differences were seen in the response rate between the socially vulnerable patients receiving either the standard or expanded CR.

The ten-year follow-up period is a major strength. To our knowledge, no previous studies have investigated the ten-year effect of a socially differentiated CR intervention. However, it must be considered that the effect of the intervention perhaps was watered down over the years and that other non-measurable competing reasons could have influenced the long-term effect.

Another strength is that even though we do not show any convincing effect of the intervention, we do show that it is possible to tailor a long-term secondary cardiovascular disease prevention which improves equality in health. In Denmark, the general practitioner is the main care provider in long-term secondary cardiovascular disease prevention. Our results show that general practitioners manage to successfully support the socially vulnerable patients except when it comes to smoking. However, the proportion of smokers increased from two to five years after after admission regardless of social status indicating that some lifestyle changes are more difficult to maintain in the long-term secondary prevention.

A weakness of the study is the external validity. It can be difficult to apply the results to countries without free access to health care and countries which do not offer reimbursement of medicine costs. However, all CR programs offered in Europe to patients suffering from CVD must be expected to comply with the recommendations by 'The European Society of Cardiology' [1].

It is also a weakness that data are based on yearly survivors if there was any selection bias in the deaths. However, it has earlier been established that no significant differences in all-cause mortality were seen at ten-year follow-up between the socially vulnerable patients receiving the standard CR and the socially vulnerable patients receiving the expanded CR [19]. Moreover, it is a weakness if the socially vulnerable patients included in the study are different than the socially vulnerable patients who were not referred to or did not participate in CR. If the socially vulnerable patients who participated were the ones with most ressources and the most healthy, there is a risk of selection bias and type-2-errors.

In addition, it seems that time plays a part in the results. One could suspect that beta-blockers were more frequently used between 2000 and 2002 and that statins were more frequently used between 2002 and 2004. Also, it seems that the development in the blood test levels could be a sign that the threshold values have been lowered over the years. Reflections concerning the non-parallel time periods in this study and the non-randomized study design have been discussed in detail elsewhere [19].

## Findings in relation to other studies

In an Italian randomized controlled trial from 2012 by Sturchio et al., the aim was to evaluate the effectiveness of an individualized management program to modify the risk profile in patients with coronary artery disease [8]. At nine-month follow-up a significant difference was seen between patients randomized to the intervention and patients randomized to standard care in relation to total – and LDL cholesterol, triglyceride, systolic blood pressure, number of smokers and adherence to beta-blockers, ACE inhibitors and statins. The intervention by Sturchio et al. [8] was significantly more effective than standard care. This could indicate that the intervention offered was more effective than the socially differentiated intervention in the present study. However, it must be emphasized that the follow-up time in the study by Sturchio et al. [8] was short. Also, there is no information in the study about the patients' social status. If only non-socially vulnerable patients participated it could cause selection bias and thus reduce the validity of the study. Moreover, it must be considered that the reason why we do not see any significant differences is that standard CR ('usual care') in Denmark has such a high quality that it may be difficult to detect any differences between a standard and an expanded intervention.

In a British systematic review and qualitative synthesis from 2014 by Rashid et al. [11], the aim was to understand the factors that promote medication persistence seen from a patient perspective. It was found that the support of family members is important, which goes well in line with the definition in this study of being socially vulnerable with little social support or not. Moreover, it was found that a good relationship between the patient and the prescribing clinician is very important, which in Denmark almost always will be the general practitioner. This supports our previous statement that general practitioners seemingly manage to support the socially vulnerable patients so well that equality in medication adherence and biological and lifestyle risk factors were improved. Furthermore, it was stated by Rashid et al. [11] that patients believe that medicine is more powerful than life-style changes. This is applicable to the results in the present study concerning smoking.

## Conclusion and implications

The present study did not find any significant long-term effects of the socially differentiated CR intervention. Overall, it was found that patients regardless of being categorized as socially vulnerable or not were adherent to prescribed medicine and that their biological and lifestyle risk factors were acceptable at follow-up. The authors acknowledge the general practitioners' effort in supporting the patients in the long-term secondary prevention and equality in health was improved in the socially vulnerable part of the study population compared to existing literature where socially vulnerable patients to a lesser extent achieve medication adherence and life style changes [12–17]. A major challenge is how to reduce the proportion of smokers in the long-term secondary prevention. where equality in health was not improved.
